# Experiences of volunteers: A volunteer-led community strategy for lung cancer awareness and mobilisation in KwaZulu-Natal

**DOI:** 10.4102/phcfm.v18i1.5141

**Published:** 2026-05-22

**Authors:** Sthabile W. Mtolo, Themba G. Ginindza, Siyabonga B. Dlamini

**Affiliations:** 1 Discipline of Public Health, College of Health Sciences, University of KwaZulu-Natal, Durban, South Africa; 2 Cancer & Infectious Diseases Epidemiology Research Unit (CIDERU), College of Health Sciences, University of KwaZulu-Natal, Durban, South Africa

**Keywords:** lung cancer, volunteers, community, awareness, mobilisation

## Abstract

**Background:**

Community health awareness is essential in ensuring communities are well-informed about different health-related challenges and promoting health-seeking behaviour. This approach could be used to address lung cancer knowledge and health-seeking behaviour, which is among the leading causes of cancer-related fatalities in South Africa.

**Aim:**

To provide an insight into the experiences of the Cancer Association of South Africa – Multinational Lung Cancer Control Program volunteers in the implementation of a volunteer-led community strategy for lung cancer awareness and mobilisation.

**Setting:**

The study was conducted in KwaZulu-Natal, South Africa, in the Durban and Pietermaritzburg cities. The Durban sites were Umlazi, Chatsworth, and South Durban Basin, and the Pietermaritzburg sites were Imbali and Sobantu.

**Methods:**

A qualitative study was conducted using focus group discussions (FGD) where a discussion guide was used. Three FGDs were conducted with 16 project volunteers. Thematic analysis was used to analyse the data.

**Results:**

Volunteers shared their insights about the strategy, understanding of their roles, skills development, community acceptance, supportive teamwork and stakeholder involvement and their preferences for community spaces versus door-to-door awareness. Challenges experienced by volunteers included community safety risks, personal safety, inadequate remuneration and intervention strategy restrictions.

**Conclusion:**

These findings highlight the need for policymakers to recognise the value of a volunteer-led intervention strategy. The challenges faced by volunteers had a direct impact on their job satisfaction and motivation. The study offers strategic insights that should inform the design and implementation of future lung cancer awareness and mobilisation programmes involving volunteers.

**Contribution:**

To inform the development of policy on volunteer-led community strategies for lung cancer awareness and mobilisation in South Africa and similar settings.

## Introduction

Cancer continues to be a universal health challenge globally, with one in nine individuals likely to develop cancer during their lifetime.^1^ The global cancer incidence rate was 20 million, with a mortality rate of 9.7 million in 2022.^2^ Furthermore, the World Health Organization (WHO) estimated new cases to increase to 29.5 million in the year 2040, with cancer mortality of 16.5 million per year.^3^ The incidence and mortality rates remain a health burden because of the disease’s associated risk factors, such as excessive tobacco use, alcohol intake and environmental and dietary factors.^1^

Prostate, lung and colorectal cancers were the most prevalent cancers in Southern Africa, according to research that focused on the 10 most frequent and deadly cancers in 56 African countries over 16 years (2002–2018).^4^ The above varies according to gender, with prostate cancer exhibiting the highest incidence in males, lung cancer affecting both males and females and colorectal cancer being more prevalent in males than in females.^4^ The African continent faces disproportionately high cancer mortality compared with high-income regions, primarily because of limited diagnostic equipment, constrained research capacity and shortages in epidemiological expertise.^1^ Lung cancer ranks among the most frequently diagnosed cancers and is the leading cause of cancer deaths both worldwide^5^ and specifically among men.^1^ The highest cause of cancer-related deaths among men in South Africa is lung cancer, which may be related to the country’s low lung cancer awareness, high rates of smoking and exposure to pollutants, among other things.^5,6^

In low- and middle-income countries (LMICs), the lack of awareness of lung cancer signs and symptoms and treatment is viewed as a barrier to early presentation and diagnosis of lung cancer.^7^ There exists minimal knowledge on lung cancer screening, hence the critical need to implement public health interventions to increase awareness of lung cancer screening, early diagnosis and treatment.^8,9^ Lung cancer-specific community awareness activities remain limited, largely because of under-resourced health promotion programmes and the scarcity of non-governmental organisations (NGOs) providing targeted lung cancer outreach, particularly in underserved communities.^10^ Research on lung cancer awareness at the community level is scarce, particularly in LMICs.^11^ An intervention study conducted in KwaZulu-Natal (KZN) in 2023 compared lung cancer knowledge of community members at baseline and post-intervention and found that more people had heard of lung cancer post-intervention compared to baseline. Before the intervention, lung cancer symptoms were less known, and the intervention increased lung cancer awareness among community members.^11^ Training community health workers (CHWs) to intervene in public health-related challenges, such as cancer, can be effective in general cancer knowledge, attitudes and beliefs among different individuals.^8^ This was evident in a community-engaged project in Georgia, United States, that Williams and colleagues undertook from 2015 to 2017, which encouraged behaviour change concerning lung cancer screening and the attempt at tobacco cessation.^8^ Community health workers continue to play a vital role in efficiently delivering healthcare services in LMICs.^10^

Because of the increased lung cancer incidence and mortality rates in South Africa between 2008 and 2018, although underreported,^12^ there exists a consistent necessity to increase lung cancer awareness and to provide educational resources on the symptoms and treatment of lung cancer to patients, healthcare providers and the general public. Therefore, the aim of this study was to provide an insight into the collaborative experiences of the Cancer Association of South Africa (CANSA) and Multinational Lung Cancer Control Program (MLCCP) volunteers in the implementation of a volunteer-led community strategy for lung cancer awareness and mobilisation in two cities in the KZN province. In this study, a strategy refers to an intervention plan designed to implement specific actions to achieve a goal, in this case, promoting lung cancer awareness and mobilisation in the study sites.

## Research methods and design

### Study design

A qualitative study utilising a Grounded Theory study design was used to understand the views, meanings and experiences of the volunteers involved in the lung cancer awareness and mobilisation volunteer-led intervention strategy. Grounded theory aims to discover a theory from data. It is appropriate for this study because it explores processes and experiences where little prior theory exists. As little is known about how volunteers navigate roles in lung cancer awareness, especially in underserved areas, grounded theory allows for generating insights directly from participants’ perspectives. This theory looks at social relationships and behaviours of groups, known as social processes. It explorers and interprets the meanings behind people’s interactions, social behaviours, and lived experiences.^13^ This study used focus group discussions (FGDs) to ensure deeper discussions about the volunteer’s experiences.

### Study setting

This study was part of a larger lung cancer initiative, the MLCCP, which spans four countries in sub-Saharan Africa: Kenya, Eswatini, Tanzania and South Africa. Multinational Lung Cancer Control Program aims to improve access to early diagnostic services for lung cancer by addressing barriers to care through collaboration with communities and Ministries of Health in these regions. In South Africa, the Cancer and Infectious Diseases Epidemiology Research Unit (CIDERU) collaborated with CANSA as an implementing partner to promote lung cancer awareness and community mobilisation. The South African component of MLCCP, funded by the Bristol-Myers Squibb Foundation and led by the University of KwaZulu-Natal (UKZN) in partnership with the Department of Health (DOH) and CANSA, was launched in KZN in 2018.

The research was conducted in KZN province, South Africa, focusing on two metropolitan cities: Durban and Pietermaritzburg. Durban sites included Umlazi, Chatsworth and the South Durban Basin (Lamontville, the Bluff and Wentworth). Pietermaritzburg sites included Imbali and Sobantu, all identified by MLCCP. The study was conducted in 2023.

### Study population and sampling strategy

The study population was made up of CANSA-MLCCP volunteers, implementing lung cancer awareness and mobilisation in the different study sites. The volunteers had been equipped with the necessary skills for lung cancer education and therefore played an important role in educating their communities. The intended sample size was 19; however, 16 volunteers were available to participate in the study. To ensure that each study site is represented, at least three or more volunteers were selected in each of the study sites. In Durban – South Basin, six volunteers were selected and available; in Umlazi, eight volunteers were selected and seven were available and one was unavailable because of health reasons. In Pietermaritzburg, five volunteers were selected, and three were available. At one of the Pietermaritzburg (PMB) sites, two potential study participants declined to take part, citing personal reasons and commitments to other work or projects. Because the nature of the study does not permit any coercion, and because they were project volunteers rather than employees, their decision not to participate was respected and accepted. Furthermore, not all the volunteers chose to participate, and this is a sign that they did not feel coerced to participate to please their supervisor.

Availability was the main criteria for inclusion, followed by period of involvement in the project and training on lung cancer. All 16 participants met the inclusion criteria to participate in the study; they had been volunteers in the project for a minimum of 6 months and were trained on lung cancer, as part of the CANSA-MLCCP project requirement. Table 1 illustrates the participant profile, followed by Table 2, which provides a detailed description of the inclusion and exclusion criteria for the participants.

**TABLE 1:** The demographic data of providers participating in the focus group discussion about the lung cancer awareness intervention.

Participant number	Gender	Age (years)	Ethnicity	Level of education	Site
1	Female	42	African person	Secondary	Lamontville
2	Female	38	African person	Secondary	Lamontville
3	Female	31	African person	Secondary	Chatsworth
4	Female	46	African person	Secondary	Chatsworth
5	Male	35	African person	Secondary	Chatsworth
6	Male	28	Mixed race person	Tertiary	Wentworth/Bluff
7	Female	37	African person	Secondary	Umlazi
8	Female	33	African person	Secondary	Umlazi
9	Female	52	African person	Secondary	Umlazi
10	Female	62	African person	Secondary	Umlazi
11	Female	32	African person	Secondary	Umlazi
12	Female	54	African person	Tertiary	Umlazi
13	Female	40	African person	Secondary	Umlazi
14	Female	30	African person	Secondary	PMB-Imbali
15	Female	40	African person	Tertiary	PMB-Imbali
16	Male	46	African person	Tertiary	PMB-Imbali

**TABLE 2:** List of inclusion and exclusion criteria of participants among the Cancer Association of South Africa-Multinational Lung Cancer Control Program volunteers.

Participants	Inclusion criteria	Exclusion criteria
CANSA-MLCCP volunteers	• Volunteers who come from the study sites. • Volunteers who have been trained on lung cancer as part of the MLCCP project requirement. • Volunteers who have been part of the project for a minimum of 6 months. • Volunteers who meet their month-to-month targets for awareness outreach in their communities.	• Community members outside of the study sites. • Volunteers who have not been trained on lung cancer as part of the MLCCP project requirement. • Volunteers who do not meet their month-to-month targets for awareness outreach in their communities.

CANSA, Cancer Association of South Africa; MLCCP, Multinational Lung Cancer Control Program.

### Data collection

The researcher facilitated FGDs to encourage volunteers to talk about their experiences with the strategy. The researcher received training in FGDs and qualitative research processes as part of her Honours Degree. The process of focus groups spanned from April 2023 to November 2023, and three FGDs were conducted. They were conducted during working hours, face to face, and the study sites’ community halls were used as the venues after permission was granted by the ward councillors. Each focus group session took between 1 h and 2.5 h. Two research assistants who received prior training and had experience in qualitative research methodologies assisted with the data collection tasks, such as note taking. The interview guide was developed from study objectives and literature on community health volunteers and cancer awareness. The topics were largely informed by the literature and the model underpinning the study. Open-ended questions explored volunteer roles, experiences and challenges with the lung cancer intervention. The guide was refined with supervisor input and minor changes after an informal pretest. Topics explored during FGDs were volunteers’ understanding of their role as educators, perceptions of existing lung cancer knowledge in the community, community acceptance and challenges experienced. The first FGD included six participants, the second had seven participants and the third group had three participants. The language used during the FGDs was a mixture of isiZulu and English. Permission was obtained from all participants to record the discussions. Anonymity was ensured by assigning numbers to each participant, which were used to indicate their responses in the transcripts instead of their names. The sessions were audio recorded, and notes were taken to ensure the researcher accurately captured the information provided by participants, ensuring the credibility of the study. The recorded discussions were transcribed and translated from isiZulu to English by a trained and experienced transcriber.

### Data analysis

The translated transcripts were analysed using the thematic analysis steps as indicated by Maguire and Delahunt.^14^ They indicate that the process of doing a thematic analysis involves using patterns found in the data and utilising them to create themes that address or focus on certain problems and subjects that are directly associated with the research.^14^ The researcher followed the steps for thematic analysis, familiarising themselves with the data while engaging and understanding the patterns. Codes were assigned, and the data were formed into themes. Lastly, the data were elaborated, interpreted and checked thoroughly. The NVivo 14-2023 software (Lumivero, Denver, Colorado State, Unted States [US]) was used to organise and classify data according to the study research questions and objectives.^15^ The verbatim quotes have been used to demonstrate the participants’ responses.

### Measures to ensure trustworthiness

Trustworthiness was established through several strategies aligned with qualitative research standards.^16^ Credibility was ensured by audio recording all participant responses and transcribing them verbatim to ensure referential adequacy, complemented by detailed field notes. Dependability was maintained through consistent documentation of the research process and the use of verbatim quotations to support the findings. To enhance transferability, thick descriptions were provided, offering detailed accounts of participants, context and procedures to enable assessment of relevance to other settings. Confirmability was supported by a clear audit trail, including audio recordings, transcripts and field notes, as well as the use of thematic analysis to guide data interpretation and mitigate researcher bias.

As a trained researcher and professional social worker who previously worked in community settings as a community mobiliser, project coordinator and a former immediate supervisor for the volunteers, I acknowledge that my prior experiences on community-based health and awareness initiatives inevitably shaped my engagement with this study. My familiarity with community mobilisation processes and volunteer-driven programmes positioned me as a partial insider to the context under investigation. While this facilitated rapport and a nuanced understanding of the setting, I remained aware that my background could influence the framing of questions, interactions with participants and interpretation of the data. Reflexivity was therefore maintained through continuous critical reflection on my positionality, assumptions and potential influence on both data collection and analysis. This included maintaining a reflexive journal to document methodological decisions, personal reflections and emerging biases, thereby enhancing transparency and ensuring that the findings remained grounded in participants’ lived experiences rather than my own preconceptions.

### Ethical considerations

This study obtained ethics approval from the UKZN Biomedical Research Ethics Committee (BREC) (Ref#: BREC/00005350/2023) and gatekeeper permission from CANSA. The informed consent process was explained, and the participants read the study information sheet. They were informed of the voluntary nature of their participation. After that, the participants signed the informed consent form.

## Results

A total of 16 volunteers participated in the FGDs. They comprised 13 females and 3 males, aged between 28 years and 62 years. As the CANSA-MLCCP volunteer database constitutes a majority of African volunteers, 15 participants were African and isiZulu speaking, and one (*n* = 1) participant was mixed race and English speaking.

The results are presented within themes and sub-themes as depicted in Table 3:

**TABLE 3:** The emergent themes and sub-themes on the provider’s perspectives of the Cancer Association of South Africa-Multinational Lung Cancer Control Program lung cancer awareness programme.

Theme	Sub-theme
1. Health education	1.1. Role identification of expanding health education 1.2. Effective health education strategies 1.3. The preferences of community spaces vs door-to-door awareness
2. Perceptions of lung cancer in the community	2.1. Lung cancer awareness at the community level
3. Community recognition and acceptance	3.1. Resistance vs acceptance 3.2. Collaborative working ethos of volunteers and stakeholders 3.3. Risks and challenges
4. Additional situational challenges	4.1. Limited access experienced by participants outside the intervention target group 4.2. Loss of community trust as a result of internal project structural inefficiencies 4.3. Inadequate remuneration

### Theme 1: Health education

#### Sub-theme 1.1: Role identification of expanding health education

The volunteers understood that the strategy aimed to educate the community through door-to-door campaigns, presentations at clinics and community health awareness events. They were aware of the importance of the intervention to ensure that communities were well informed about lung cancer:

‘… we are sent out to teach people, yes, and lung cancer is a disease that is there, people do not have enough information about lung cancer.’ (P2, Female, 38 years)

A quarter of participants expressed how the acquired skills, excellent training and knowledge gained as part of the strategy encouraged them to work confidently and remain motivated. Some volunteers shared that before the training, they possessed a minimal understanding of cancer. Hence, the training was appropriate to create confidence in their engagement with the community:

‘I have that knowledge, so being part of this project helped a lot in giving us information to share in the community.’ (P10, Female, 62 years)

‘It is easy; the more you learn, the more you gain knowledge, and you continue to share the required knowledge.’ (P12, Female, 54 years)

All 16 volunteers also reflected on gaining more experience through learning and raising awareness in their communities. In particular, focusing on awareness increased their self-confidence. Although they described the door-to-door intervention as a challenging strategy, they nonetheless learned techniques for effectively communicating with different people. In particular, they cited factors such as how to speak, their behaviour and their attitude during home visits. It was evident from P4, who related how being self-aware of her own decorum when engaging with the community is facilitative. This also influenced how they were perceived as individuals and their work:

‘… when you are talking to someone, you should not have a cold face, we call it a “stubborn face.” You must be free and forget about your problems at home. You see, when you are at work, concentrate on your work so that everything can go well.’ (P4, Female, 46 years)

‘… When you accept her with warmth and openness, the patient can open up and talk to you.’ (P9, Female, 52 years)

Another pertinent component was how their knowledge about lung cancer also increased their understanding of other illnesses, such as tuberculosis (TB). This further emphasised how their strategy was to keep community members informed of the similarities between lung cancer and TB symptoms:

‘… as we know the signs and symptoms of lung cancer are similar to the TB ones, so when we tell them about the similarities, they benefit a lot from this information.’ (P3, Female, 31 years)

#### Sub-theme 1.2: Effective health education strategies

Unity and teamwork were identified as salient in adding confidence to the strategy. Their race, age and gender added to the dynamic of appreciation of such differences in the spirit of teamwork and collaboration. Hence, the techniques of working in pairs or groups when visiting people’s homes and planning and attending awareness events in public gatherings and clinics together were prominent features noted by participants. Such diversity created a supportive ethos among them as volunteers, as noted:

‘Yes, we were united, and our leader taught us respect. Our foundation was strong, and even when CANSA took over, our foundation was already strong. We started working with Indians and the Mixed race, but we were one. We enjoyed working together.’ (P14, Female, 30 years)

P15 in particular offered how teamwork maximised sharing duties when engaging with the community. This also included the division of labour, which ensured that different roles were played to ensure the effectiveness of the intervention:

‘When you are a pair, one will distribute the pamphlets whilst the other is standing before the people.’ (P15, Female, 40 years)

#### Sub-theme 1.3: The preferences of community spaces vs door-to-door awareness

Beyond the door-to-door strategy, community spaces were another effective place to engage community members more deliberately. Community spaces are versatile areas that cater to a wide range of activities for individuals. In this study, community spaces refer to places where individuals seek health and medical attention, such as clinics, as well as attend community events to learn more about various health-related topics:

‘The way the strategy works, so far, I am happy about it because we go door-to-door. We talk about it at the clinics. We talk at community meetings. It is working well because if they are not in the clinics, we reach them at home. If not at home, then they reach us at the meetings.’ (P2, Female, 38 years)

Volunteers in the Durban sites noted the effectiveness of community education in clinics and community events, whereas in the Pietermaritzburg sites community members were distracted at clinics and events; therefore, educating them in their homes was preferable. P15 from PMB noted:

‘People come to the clinic for different reasons and, therefore, while you are talking, they are distracted by clinic activities, like being called next in the queue, so they end up losing the information that they were meant to receive. Whereas when they are at home, you sit down with them, they give you attention, and you leave them with enough information.’ (P15, Female, 40 years)

On the other hand, a Durban volunteer had this to say:

‘What boosted us is having clinics. When we go to the clinics, we can see a lot of people. Whereas, when you go by yourself door-to-door, you do not know the risks.’ (P9, Female, 52 years)

### Theme 2: Perceptions of lung cancer in the community

#### Sub-theme 2.1: Lung cancer awareness in the community

According to many volunteers, it became evident that community members were less knowledgeable about lung cancer. They conceded that their involvement was important in promoting health awareness in their communities:

‘In my opinion, it is good because it has helped many people, as a lot of people did not have much knowledge about it in the community, and now, they have information.’ (P4, Female, 46 years)

Some misconceptions were also noted:

‘Some of the beliefs that community members had was that a person would not get lung cancer if they were not smoking.’ (P11, Female, 32 years)

This statement may be partly true because eliminating smoking can reduce the risk of lung cancer. However, there is less awareness about other factors associated with the risks of lung cancer, such as exposure to secondhand smoke, asbestos, pollution and a family history of lung cancer.

Another participant also noted the lack of early detection:

‘They live with the disease for a long time until it reaches the last stage, and they cannot get any help, so us going door-to-door teaching them, they will get help.’ (P5, Male, 35 years)

Another related observation was the willingness of community members to learn about lung cancer, while others demonstrated minimal interest as shared by P6. The latter left volunteers demotivated:

‘Some people would tell us that they do not want to know about lung cancer, but most people were genuinely interested and wanted to know more, including how to get screened because their health is important to them.’ (P6, Male, 28 years)

Some participants suggested that interest is only piqued when the community members are directly affected. P12 shared that:

‘In the communities we come from, individuals tend to pay attention to something, including health-related matters when someone gets seriously sick or dies, that is when they see the seriousness of it.’ (P12, Female, 54 years)

### Theme 3: Community recognition and acceptance

#### Sub-theme 3.1: Resistance vs acceptance from the community

As the strategy is community based, the focus was on promoting lung cancer awareness, screening for symptoms and providing support to those affected by cancer. The level of acceptance was determined by factors such as understanding of the intervention strategy. It was, nonetheless, evident from P4’s comment that most communities accepted the volunteers’ entry into their communities, especially when they understood their roles and responsibilities:

‘… from when we started working as volunteers …, we were welcomed well in the community, and the challenges were that many people did not understand.’ (P4, Female, 46 years)

Although all volunteers had identification, such as name tags and t-shirts, and the community leaders were aware of the intervention strategy, because of it being a new project, the community members sought clarity as they needed a better understanding of it:

‘They asked where we came from, who hired us, and why us, but as time went on, we worked well together.’ (P14, Female, 30 years)

However, a sense of resistance was evident, especially in the early stages of the strategy, where community members were sceptical about letting volunteers into their homes. A participant shared that:

‘There were those challenges that people did not want to open their homes, and they would say that they were busy, but we would not force them to listen to us.’ (P15, Female, 40 years)

Although at first, the community was sceptical about the volunteers, they eventually welcomed them, which afforded them a sense of value. This acceptance motivated the volunteers to continue working more diligently in their respective communities. There was a general sense of fulfilment as noted by P2, as they were playing an important role in their communities. There was a sense of trust and acceptance from the community:

‘… we have helped a lot of people without realising it because we think we are just working in the community; I am happy so far because we are getting positive responses from the community.’ (P2, Female, 38 years)

#### Sub-theme 3.2: Collaborative working ethos of volunteers and stakeholders

According to most participants, they had a positive and productive relationship with the stakeholders in their communities. The community leaders and ward councillors supported the strategy. Clinic managers and staff were welcoming, as noted by P7 and P10. Many volunteers reflected that they could provide awareness in the clinics with the support of the staff, inferring that they work collaboratively. Their cancer knowledge and expertise were well recognised by other stakeholders in the community. Consequently, they were invited to mass health awareness community events to raise awareness about lung cancer. Describing the relationship between volunteers and community stakeholders, as commented:

‘Our community councilor does not have a problem. He is supportive, and he even offers to help you in the way you want so that you can succeed.’ (P7, Female, 37 years)

‘Even at the clinics, the sisters and matrons we came across do not have a problem; they welcome us with open arms, and they are happy to see us. I remember the manager at D clinic was even advising us about other things that we could tell the patients.’ (P10, Female, 62 years)

#### Sub-theme 3.3: Risks and challenges

Although volunteers were welcomed by community members, issues of safety were noted by most volunteers when making home visits. They were aware of the risks, but the willingness to bring about change in their communities kept them motivated, as noted by P7. In particular, safety was a concern for the female volunteers, who explained that home visits were worrisome, especially when alone; hence, they preferred working in pairs for door-to-door visits:

‘As much as we continue to work because we want our people to learn, it is not 100% safe. We can enter (the house) as women but it’s scary. That is why when we go to people’s homes, we sometimes insist on sitting outside even if it is cold as we know that entering the house is not safe, and we always make sure that we go in pairs.’ (P7, Female, 37 years)

In particular, P13 established that she remained motivated because of her passion for her work. However, some volunteers shared that till that point, there had not been any incidents that exposed them to any type of danger. It seemed from P13 that within the present SA environment, it was acceptable for them to be apprehensive:

‘Firstly, with this door-to-door, we go to homes that we do not even know who stays there or what, that is also risky, but we go because we love our jobs.’ (P13, Female, 40 years)

Another concomitant factor was the prevailing political and crime-related context. All PMB volunteers shared that they felt unsafe during home visits and working within the community. Volunteers explained that such safety issues used to happen before and during political activities taking place, such as voting seasons. Their communities were affected by politically related murders. P15 shared these safety and security issues:

‘We are not safe in our communities; people are being killed due to crime and political unrest. As we are out in the community we are scared for our lives.’ (P15, Female, 40 years)

### Theme 4: Additional situational challenges

#### Sub-theme 4.1: Limited access experienced by participants outside the intervention target group

Although positive experiences were noted, they were also confronted by various challenges. The intervention strategy included specific communities of the larger MLCCP study. This meant that some communities were not benefiting from the education and awareness. This situation, especially, became an issue when volunteers started visiting local clinics to provide awareness of lung cancer and assess community members presenting with signs and symptoms that required further screening. They identified community members who were not from the areas that the project targeted. It was challenging for them, as described by P11, whose mandate of work site excluded other locations although the individuals attended the same clinic:

‘… it becomes hard if we find out that they are not in the ward that we are covering.’ (P11, Female, 32 years)

However, volunteers added that when reported to their coordinator, exceptions were made for the few community members who needed the service but were residents from other wards. Additionally, working closely with the UKZN-MLCCP clinical team, the volunteers informed other community members of the details of the upcoming community screening events near them (i.e., dates, clinic venues and times) so that the community members from other areas could have access to such services if necessary.

#### Sub-theme 4.2: Loss of community trust as a result of internal project structural inefficiencies

The MLCCP also experienced challenges in reaching bilateral agreements with the provincial Ministry of Health. These challenges resulted in role confusion, missed expectations and delays in the delivery of some interventions, such as screening interventions conducted by the programme clinical team and volunteers. However, even though contingencies existed to mitigate these issues, which communities were informed of, the challenges persisted. The volunteers shared that the community members lost trust and confidence in them when it came to screening. The community members accused the volunteers of empty promises:

‘Even if it is fast now, we already have a bad reputation in the community because we screened people, but they waited, and the X-ray did not come. I have four patients who passed away who never went for an X-ray, and when they became very sick and went to the hospital, the family told me that they found out that it was cancer that I was teaching them about.’ (P7, Female, 37 years)

‘Hey, one man said we were scammers … we became the bad guys in the community because we didn’t come back, especially with screening. It did not come according to the promises we made to the people.’ (P16, Male, 46 years)

However, some of these challenges were overcome towards the end of the project:

‘There was a lack of trust, but now they can see that it is faster, as it is said that the mobile X-ray would come monthly.’ (P7, Female, 37 years)

#### Sub-theme 4.3: Inadequate remuneration

The stipend that the volunteers received created discontent. They shared that they had not had an increment in the three (*n* = 3) years of the project. It appeared that their concerns were not being heard, as it seemed like there was little commitment from the authorities to ameliorate the situation. In addition, volunteers had an increased workload over the years, but the stipend remained the same, which is articulated:

‘The stipend is little. It is the main problem because the workload is heavy on us and it’s pilling up, yet the money is less.’ (P14, Female, 30 years)

Most volunteers shared their concerns about the inadequate and non-market-related compensation, as noted by P15. Their portfolio of duties included clinic visits, planning and conducting awareness events, door-to-door visits and care and support. They were not remunerated for costs, such as transport expenses. The volunteers suggested that in future projects, expenses, such as travelling costs, where necessary, be properly considered in the planning stage of the project to avoid such challenges:

‘It is not much, and it does not cover most of our needs. We have so many needs, and our families are expecting help from us.’ (P15, Female, 40 years)

As most volunteers stated their dissatisfaction with low stipends, they also mentioned that their payments were occasionally late or not delivered on time as specified in their contracts. One participant states:

‘We get payment very late, and the money is less yet the work is so much.’ (P7, Female, 37 years)

## Discussion

For this study, awareness is defined as the promotion of education on lung cancer to increase knowledge of lung cancer signs and symptoms, causes and risk factors.^17^ This involves recruiting and training community members (CANSA-MLCCP volunteers) to educate the public about lung cancer. Community health awareness is fundamental in ensuring increased knowledge about different health issues.^18^ The volunteer-led intervention strategy on lung cancer awareness and mobilisation aspired to educate local communities about lung cancer to improve public awareness of the signs and symptoms, while encouraging health-seeking behaviour, thus, contributing to decreased lung cancer mortality and morbidity.

Volunteers recognised their responsibility as disseminators of awareness and knowledge to assist their communities in remaining informed about lung cancer. They were confident about their role. Cancer Association of South Africa successfully provided training to enhance their skills to educate the community. Training was one of the motivating factors to transfer health knowledge to others. Dlamini and colleagues support that educating workers of a similar calibre (CHWs) to increase their skills, and knowledge improves their confidence and willingness to work for the community.^10^ Volunteers valued the training and knowledge received, and their eagerness to learn more was evident in this study. Therefore, further training and skills were expressed as an important aspect of the strategy to ensure that they are fully equipped with the knowledge, skills and resources they need to be confident in their work. Recent studies report limited training as one of the major concerns, where workers of a similar calibre indicated a desire for ongoing refresher training to keep them up to date.^19^

An important motivation for volunteers was the positive relationship they shared with their coworkers. A recent South African study found that unity and teamwork facilitated the CHW’s work performance.^19^ Similarly, in our study, volunteers supported one another, learned from each other and maintained an ongoing team spirit. An Ethiopian study revealed that positive relationships with coworkers were one of the key factors for job satisfaction for CHWs.^20^ Our study results suggest the importance of a supportive environment amongst team members in such intervention strategies, encouraging teamwork and improving the work performance of volunteers.

Community acceptance and positive recognition were important factors for volunteers. Volunteers were well recognised in the community, and this kept them motivated. This was noted as the majority of community members expressed their readiness to learn from volunteers about lung cancer. Respect, trust and recognition from the community are important facilitators for volunteers, as this ensures successful community health volunteers’ involvement in community interventions.^21,22^ Community participation and ownership play a significant role in the community health volunteers’ work motivation.^23^ The relationship shared with community stakeholders was one of the highlights for volunteers; support from community leaders, supervisors and healthcare professionals kept them motivated. Dageid et al.^24^ found that good networks ensure assistance and support for volunteers working in communities. Good social relations improve access to resources that marginalised communities may need.^24^ Internal project structural inefficiencies, challenges and delays in promised services for the community contributed to the uneasiness of volunteers because of low trust, as they were failing their communities. Such findings suggest the need for a positive relation between community trust and service delivery, that for communities to accept and trust intervention programmes, there needs to be adequate service delivery, which motivates a good relationship and support between communities and health care community interventions, that is CHWs. Although volunteers were responsible for educating the public about lung cancer as part of the study, another important component of the larger MLCCP study involved facilitating lung cancer screening. Consequently, during their educational sessions, volunteers informed community members that lung cancer screening, specifically X-ray screening drives, would be made available for those who wished to participate. These screening activities were ultimately implemented; however, they did not occur exactly as initially communicated by the volunteers, particularly regarding the anticipated timeframe.

The nature of community health work requires community visibility through home visits and community social spaces, such as community events. In our study, volunteers visited people in their homes, and a safety concern was reported; however, volunteers expressed their level of understanding of the nature of their jobs. Female volunteers felt more at risk visiting the different households; therefore pairing up during home visits was the solution for them. Greater effort is required to protect and ensure safety in our communities. South Africa is reported to have the third-highest crime rate worldwide.^25^ Community health workers in a study conducted in the Eastern Cape, South Africa, shared their concerns about their personal safety, mentioning being bitten by dogs while doing home visits, making access to some houses impossible and unsafe in forest areas.^19^ Although the issue of risk because of dogs was brought up in our study, it was not a major safety concern. Volunteers felt unsafe not knowing who was in the houses they were visiting, and they were worried about their lives being at risk because of high crimes in their communities, such as murders, rape and politically related killings. Literature reveals that safety is an important factor for volunteers working in different communities, as this increases the protection that they require in their jobs, ensuring less danger or risk.^26^ The DOH and local NGOs should consider implementing comprehensive strategies to ensure the protection and safety of volunteers. This may involve collaborating with community policing forums, as well as equipping volunteers with safety devices such as panic buttons and pepper sprays. Additionally, establishing clear protocols for emergency response and communication can further enhance the safety measures for volunteers.

Volunteer’s preferences for community spaces compared to door-to-door awareness differed according to the different study sites. PMB volunteers preferred door-to-door visits, as they were less distracted compared to community spaces such as clinics. They stated that during home visits, individuals were more focused, giving individuals an opportunity to ask questions, raise concerns and express themselves freely. Participants in PMB also reported on the community members’ reluctance to use the normal health service offerings because of their perceptions of long queues and waiting times and poor staff attitudes in the public healthcare facilities.

Durban volunteers, on the other hand, noted the effectiveness of education in clinics and community events because of the mass accessibility of community members, they shared that during home visits sometimes people are not available, they are out busy with their daily activities, such as work, shopping or could be at home but would be busy with their house chores, thus less interest on learning. It may have been beneficial for volunteers to request appointments at times when community members were less busy at home. This possibility was not investigated further in this study. However, it could be a valuable lesson learned to schedule appointments based on the availability of the community members. Ryman et al.^22^ found that CHWs viewed home visits as more meaningful and effective, as these visits enabled them to deliver basic health care directly to households and helped reduce barriers like long trips to clinics.

The volunteers’ negativity towards the strategy stemmed from issues related to dissatisfaction with their stipend. The issue of a low stipend, with no increase for over 3 years, was reported by volunteers as demotivating. Woldie and colleagues refer to community health volunteers as volunteers who do not receive a regular salary or hold a formal position in the health system,^23^ whereas CHWs are defined as a lower-skilled calibre of healthcare workers who are paid a minimum wage.^27^ Aseyo and colleagues note that an incentive for work done is deemed important for the satisfaction and individual needs of CHWs. Regular, adequate financial compensation for community health volunteers is key to motivation, retention and programme effectiveness. Generally, the scope of volunteers ranges from paid volunteers to those receiving a stipend and those who voluntarily support their communities without remuneration.^28^ In South Africa, the remuneration of CHWs depends on whether they are employed in the public sector or subcontracted through NGOs; however, there is no legislation governing the payment of CHWs in SA.^28^ A study in Ethiopia found lack of remuneration to be a demotivating factor for community health volunteers and one of the critical causes of poor retention of volunteers.^20^

According to Made et al.,^29^ planning and execution of community health interventions entail important factors such as budgets and financial expenditure, and depending on the health project, volunteers receive stipends for the volunteer work that they do. A South African study reported that community health volunteers reported that while wishing to be remunerated, they still find satisfaction in the community recognition that came with being a volunteer and the development of new skills and knowledge.^24^ Although dissatisfied with the compensation, volunteers viewed other aspects, such as altruism to work for the community, knowledge and skills acquired, and community acceptance and support, as benefits of being part of the intervention strategy. However, such findings suggest that volunteers desire a more reasonable remuneration to cover their basic needs.

### Strengths and limitations

This study employed qualitative methods, facilitating a comprehensive understanding and articulation of the participants’ experiences. Data were collected through FGDs, which facilitated a deeper understanding of shared life experiences within the framework of the volunteer-led intervention strategy. The insights shared by the participants highlight the value of qualitative methodologies, which can uncover rich, nuanced experiences that might not be captured through quantitative approaches. Therefore, qualitative research is crucial to supplement quantitative studies and provide a comprehensive understanding of the volunteer-led intervention for lung cancer awareness and mobilisation at the community level, particularly within the South African context.

The study yielded significant findings; however, it is essential to acknowledge that these results cannot be broadly applied because of the intrinsic characteristics of qualitative research methodology. Furthermore, the research was confined to select MLCCP study sites, which means that only a limited number of areas within the KZN province were represented. This geographical restriction may have constrained the diversity of experiences and opinions captured in the study.

## Conclusion

It is also important for public health policy to inform the development of policy on volunteer-led community strategies for lung cancer awareness and mobilisation in South Africa and similar settings. The study provided strategic information to inform future programme implementation to consider the challenges faced by volunteers concerning community acceptance, limited skills and resources, remuneration and community work safety. Support from the community, stakeholders, employers and teamwork contributes to the retention of community health volunteers. Community trust and acceptance are important factors for community health intervention; therefore, measures to maintain this need to be constantly practised. It is imperative for public health policy to contemplate approaches to alleviate the challenges documented in both our data and existing literature, concerning inappropriate remuneration, ongoing training and the personal safety of volunteers involved in community health work.
